# E2F6 is essential for cell viability in breast cancer cells during replication stress

**DOI:** 10.3906/biy-1905-6

**Published:** 2019-10-14

**Authors:** Inam Jasim LAFTA

**Affiliations:** 1 Department of Microbiology, College of Veterinary Medicine, University of Baghdad, Baghdad, Iraq

**Keywords:** Breast cancer, E2F6, replication stress, oncogene

## Abstract

E2F6 is a member of the E2F family of transcription factors involved in regulation of a wide variety of genes through both activation and repression. E2F6 has been reported as overexpressed in breast cancers but whether or not this is important for tumor development is unclear. We first checked E2F6 expression in tumor cDNAs and the protein level in a range of breast cancer cell lines. RNA interference-mediated depletion was then used to assess the importance of E2F6 expression in cell lines with regard to cell cycle profile using fluorescence-activated cell sorting and a cell survival assay using (3-(4,5-dimethylthiazol-2-yl)-2,5-diphenyltetrazolium bromide (MTT). The overexpression of E2F6 was confirmed in breast tumor cDNA samples and breast cancer cell lines. Depletion of E2F6 in the breast cancer cells reduced cell viability in MCF-7, T-47D, and MDA-MB-231 cells. There was little effect in the nontumor breast cell line MCF-10A. The deleterious effect on cancer cells was greater during replication stress, leading to an increase in the proportion of breast cancer cells with sub-G1 DNA content. These results suggest that E2F6 might be essential for the survival of breast cancer cells experiencing replication stress, and therefore it could be a target for combined therapy.

## 1. Introduction

The *E2F* genes encode a family of nine transcription factors with one or more conserved DNA binding domains. They bind promoters as either homo- or heterodimers and target distinct and overlapping promoters to regulate gene expression (Trimarchi and Lees, 2002; Attwooll et al., 2004). Proteins E2F1 through E2F6 also contain a conserved domain responsible for binding to dimerization partner proteins (de Brucin et al., 2003; Di Stefano et al., 2003). The established paradigm from in**vitro studies is that a network of signals converges on retinoblastoma (Rb) and related proteins to cause E2F-dependent changes in transcription, which regulate progression through the cell cycle and thus contribute to cell proliferation. The E2F family members have also been shown to control the expression of genes implicated in DNA replication, DNA damage repair, cell fate, and mitosis (Trimarchi and Lees, 2002; Attwooll et al., 2004; Cam et al., 2004; Dimova and Dyson, 2005; Bieda et al., 2006; Buttitta and Edgar, 2007; McClellan and Slack, 2007; Zalmas et al., 2008; Chen et al., 2009). Pathways regulated by E2Fs are clearly implicated in breast cancer and can be overexpressed in drug-resistant tumors (Johnson et al., 2016). E2Fs are also implicated in upregulating genes whose expression correlates with metastasis and poor prognosis in breast cancer (Thomassen et al., 2008; Thangavelu et al., 2017). Previously, it was thought that E2F1 to E2F3a and E2F3b act only as activators of transcription while E2F4 through E2F8 act as repressors; this has now been challenged and the activation or repression function is likely to be tissue- and context-dependent (Chong et al., 2009; Lee et al., 2011; Weijts et al., 2012). 

E2F6 differs from other family members because it lacks the C-terminal sequences required for transcription activation and interaction with pocket protein family members such as Rb protein (pRb) (Morkel et al., 1997; Gaubatz et al., 1998; Trimarchi et al., 1998). E2F6 is not regulated by pRb or its homologues p107 or p130, but it forms a complex in proliferating cells with polycomb group proteins, counteracting the activity of the other E2F complexes and causing transcriptional repression of E2F responsive genes (Trimarchi et al., 1998, 2001; Ogawa et al., 2002; Attwooll et al., 2005). E2F6 is, therefore, thought to be a pocket protein-independent transcriptional repressor (Morkel et al., 1997; Cartwright et al., 1998; Trimarchi et al., 1998, 2002). E2F6 is expressed throughout the cell cycle but accumulates during G1, reaching a peak at the G1/S transition (Kherrouche et al., 2004). During the S-phase, E2F6 interacts with E2F target genes that are activated at G1/S, thus restricting their expression and promoting progression through the cell cycle (Giangrande et al., 2004; Bertoli et al., 2013). 

Although *E2F* genes are not frequently mutated in cancer, amplification and/or dysregulation of E2F expression is correlated with abnormal expression of tumor suppressors and malignancy (Polanowska et al., 2000; Fang and Han, 2006; Chen et al., 2009). There is known redundancy for E2F proteins in normal cell proliferation (Gaubatz et al., 2000; Danielian et al., 2008; Tsai et al., 2008; Zalmas et al., 2008), but it has been suggested that tumors may become addicted to specific E2F activators during oncogenic proliferation (Chen et al., 2009). A logical prediction would be that in tumors there could be overexpression of E2F activators (functioning as oncogenes) and loss of E2F repressor activity (tumor suppressors). However, this does not always appear to be the case, with several studies suggesting a function for E2F4–8 (considered to be repressors) in promoting tumorigenesis (Polanowska et al., 2000; Reimer et al., 2006; Bindra and Glazer, 2007; Endo-Munoz et al., 2009; Umemura et al., 2009). 

E2F6 was reported to be overexpressed in a series of ER-negative/P53-positive breast carcinomas (Palacios et al., 2005). Furthermore, expression of a potential negative regulator of E2F6 microRNA-185 (miR-185) is downregulated in triple-negative breast cancer (i.e. negative for estrogen ER, progesterone PgR, and human epidermal growth factor receptor HER2/ERBB2) and associated with poor prognosis (Tang et al., 2014). Here we confirm the overexpression of E2F6 in breast cancers and also test the idea that E2F6 overexpression could be important specifically to the survival of breast cancer cell lines. 

## 2. Materials and methods

### 2.1. Tissue array

Gene expression was analyzed in tumorous and normal breast tissues using the TissueScan Breast Tissue qPCR array (Cat. No. BCRT302, Origene Technologies, Rockville, MD, USA). This tissue scan is composed of a panel of 43 cDNAs from breast tumor tissues representing four different TNM stages of breast cancer and 5 cDNA samples from adjacent normal breast tissues. A detailed pathology report is provided for all the purchased cDNA samples, which can be reviewed on the website of the aforementioned company. 

### 2.2. Mammalian cell lines

All cell lines were obtained from ATCC, except Jurkat cells, which were a gift from Professor Holley, University of Sheffield. MCF-7, MDA-MB-231, MDA-MB-468, and T-47D cell lines were grown in DMEM containing 4.5 g/L glucose with L-glutamine, 10% FCS (Seralab) and 1X nonessential amino acids (Bio Whittaker). Jurkat cells were grown in RPMI 1640 (Lonza) containing L-glutamine, 10% FCS and 1X nonessential amino acids. MCF-10A cells were grown in DMEM containing 4.5 g/L glucose with L-glutamine with the addition of 1X nonessential amino acids, 5% horse serum (Invitrogen), 10 µg/mL insulin (Sigma-Aldrich), 0.1 µg/mL cholera toxin (Calbiochem), 10 µg/mL epidermal growth factor (EGF; Sigma-Aldrich), and 50 µM hydrocortisone (Sigma-Aldrich). All cell lines were used within 20 passages and regularly checked for *Mycoplasma*.

### 2.3. qRT-PCR

**Figure 1:**
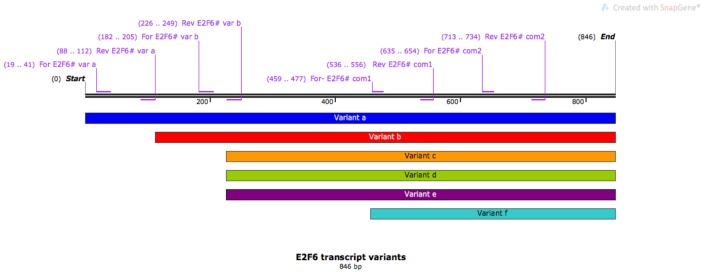
Scheme depicting positions of the amplification primers in E2F6 cDNA. Six transcript variants (a to f) of the E2F6 gene are shown parallel to its cDNA. Among the primers, two sets were common to all the transcripts and termed E2F6# com1 and E2F6# com2. While one primer set was specific for variant a (E2F6# var a), the other primer pair was specific for variant b (E2F6# var b).

Total RNA was extracted using the RNeasy Mini Kit (QIAGEN). cDNA was obtained using 1 µg of total RNA and the Applied Biosystems High Capacity cDNA Reverse Transcriptase Kit (Thermo Fisher Scientific), and 5 µL of cDNA was mixed with SensiMix SYBR (Bioline) and 10 mM primers. Primers were designed to amplify cDNA transcripts of 100–150 bp and were ordered from Eurofins. As a negative control for quantitative real-time polymerase chain reaction (qRT-PCR) assay, two sets of Fli1 primers were included to amplify the *FLI1 *gene, which is a protooncogene known to be upregulated in acute myeloid leukemia but underexpressed in breast cancer cell lines; therefore, it was used here to demonstrate that gene expression was not upregulated across the genome in breast cancer and also to validate qPCR results. The primers and the purpose of their use are shown in the Table below. Figure 1 shows a scheme that depicts positions of the amplification primers in E2F6**cDNA.

**Table:** The sequences of amplification primers and the purpose of their use.

Primer name	Purpose	Sequence 5’-----------3’
E2F6# com1	Common to all variants	For-GGAAGATGCTTTGGATGAG Rev-GATAGGTCACATATGCTAGTC
E2F6# com2	Common to all variants	For-TTCCAGCTCCCAGAGAAGACRev-TTACTGGTCTGACCCTGCTCCA
E2F6# var a	Specific to variant a	For-GCGAGGAAGTTACCCAGTCTCCTRev-ATGGCAGCAGGCCCTCCACGTTGAT
E2F6# var b	Specific to variant b	For-CCAGCGATACATCAAAACGAGGTCRev-ATGGATCTTGTCAGATCTGCTCCC
β-Actin	Internal control	For-CAGCCATGTACGTTGCTATCCAGGRev-AGGTCCAGACGCAGGATGGCATG
18S	Internal control	For-AGAAACGGCTACCACATCCARev-CACCAGACTTGCCCTCCA
FLI1#1	Negative control	For-GAATTCTGGCCTCAACAAAAGRev-CCCAGGATCTGATACGGATCT
FLI1#2	Negative control	For-ATCCAGCTGTGGCAATTCCTRev-CATCGGGGTCCGTCATTTTG

qRT-PCR was performed using the Corbett Robotics Rotor-Gene 6000 (QIAGEN). The cycling conditions were 95 °C for 10 min followed by 40 cycles of denaturing at 95 °C for 15 s and annealing at 58 °C for 15 s and finally 30 s at 72 °C for extension. Using Rotor-Gene 6000 software, the C_T_ (the threshold) value was determined for each cDNA sample in every reaction. Relative gene-expression value was calculated according to Livak and Schmittgen (2001) using the following formula: ΔCT = CT target gene – CT endogenous reference gene, using β-actin for tissue arrays and 18S for cell lines as the reference gene. The fold change compared to the reference sample was then calculated using the delta-delta CT method, also known as the 2–∆∆CT method, where ∆∆CT = ∆CT (tumor sample) – ∆CT (normal sample) (Livak and Schmittgen, 2001). In the case of cell lines, MCF-10A was taken as a reference sample. For tissue array samples the median value of 5 normal tissue samples was used as a reference sample. 

### 2.4. si-RNA transfection

**Figure 2:**
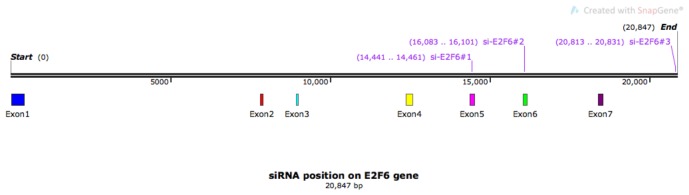
Positions of the si-RNAs on the E2F6 gene. The gene map shows the distribution of exons on it with the locations of the three si-RNAs specific to E2F6 targeting different regions.

Cells were reverse-transfected with 20 nM (final concentration) small interfering RNAs (si-RNAs) using Dharmafect 4 reagent (Dharmacon) and following the manufacturer’s instructions. All si-RNAs were purchased from Eurofins. Sequences were as follows: si-E2F6#1 AAGGAUUGUGCUCAGCAGCUG (Oberley et al., 2003), si-E2F6#2 AGUUAAAGCUCCAGCAGAA, and si-E2F6#3 CUUAAGAAGUGCUCAAUAA (Lafta, 2016). Figure 2 shows the positions of the si-RNAs on the *E2F6 *gene.

### 2.5. Survival assay (MTT)

Following si-RNA transfection, cells were left for 96 h, after which time 1 mg/mL MTT (3-(4,5-dimethylthiazol-2-yl)-2,5-diphenyltetrazolium bromide; Thermo Fisher) was added to each well and the cells left for 3 h at 37 °C. The medium was then aspirated off and replaced with 200 µL of DMSO (Fisher Scientific), and the OD was measured at 595 nm on a plate reader. The experiment was repeated in triplicate on at least 3 separate occasions and the mean and standard error of the mean were calculated.

### 2.6. Cell cycle analysis

Twenty-four hours after transfection with si-RNA, 2 mM hydroxyurea (HU) was added to the cells, which were left for a further 24 h. Cells were then fixed in 70% methanol and left overnight at –20 °C. After washing in phosphate-buffered saline, cells were stained with propidium iodide/RNase A solution (50 mg/mL PI, 100 mg/mL RNase A) for at least 30 min. Samples were analyzed by flow cytometry (Becton-Dickenson FACSort, 488-nm laser). Quantification of the percentage of cells in individual cell cycle phases was performed using the CellQuest IX flow cytometry software package. Each experiment was repeated on 3 separate occasions and the mean and standard deviation were calculated.

### 2.7. Western blotting

Cells were lysed in RIPA buffer in the presence of 1X protease and phosphatase inhibitor cocktails (Roche). An aliquot of 30 µg of total protein was run on sodium dodecyl sulfate polyacrylamide gel electrophoresis (SDS-PAGE) gel and transferred to Hybond ECL membrane (GE Healthcare). This membrane was immunoblotted with antibodies against E2F6 (1:1000, Santa Cruz), β-actin (1:5000, Sigma), and β-tubulin (1:5000, Sigma), each diluted in 5% milk and incubated at 4 °C overnight. After the addition of the appropriate horseradish peroxidase-conjugated secondary antibody and further washes, the immunoreactive protein was visualized using ECL reagents (GE Healthcare) following the manufacturer’s instructions. Protein on western blots was quantified relative to actin or tubulin using ImageJ software.****Tubulin was used as a loading control to compare expressions between cell lines and actin used within each cell line.

### 2.8. Statistical analysis

Results were determined to be normally distributed using the Shapiro–Wilk test for normality prior to analysis with a paired 2-tailed Student t-test or an unpaired 2-tailed Mann–Whitney U test as indicated. P < 0.05 was considered representative of data that were significantly different. GraphPad Prism 7 software was used for analysis of all data.

## 3. Results 

### 3.1. E2F6 expression is greater in breast cancer than normal tissue 

**Figure 3:**
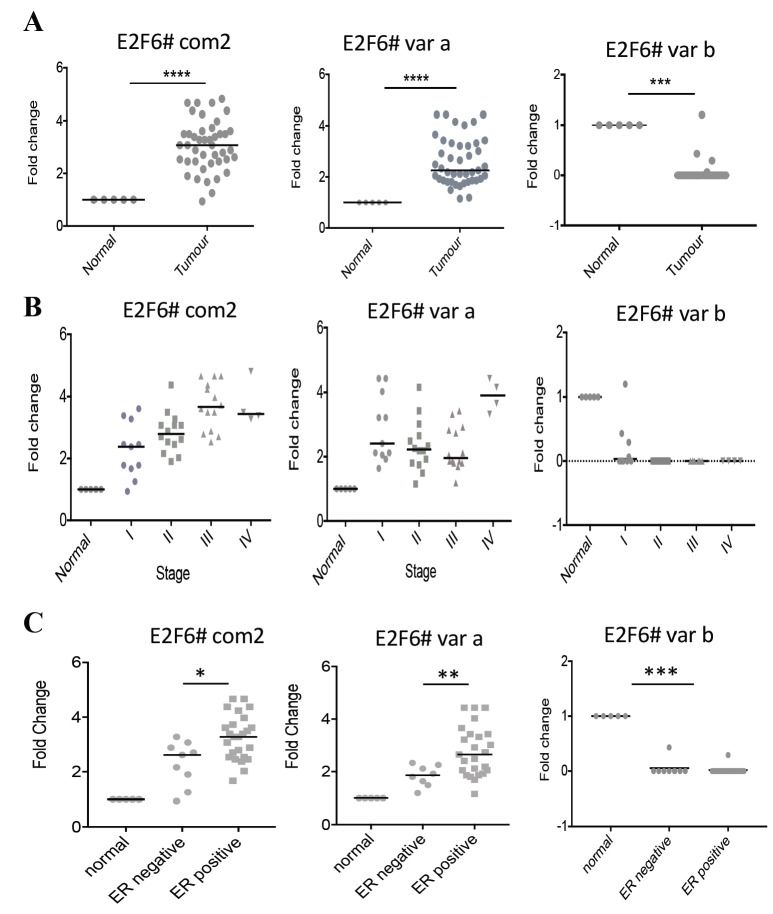
E2F6 expression is increased in breast cancer compared to normal breast tissue: (A) 48 breast tissue cDNAs were used to investigate the fold change in cDNA levels of E2F6 using three sets of primers: E2F6# com2 (common to all E2F6 variants), E2F6# var a (specific for variant a), and E2F6# var b (specific for variant b). Results were first normalized to β-actin and then shown relative to the median E2F6 expression level across 5 normal breast cDNAs. The same data are displayed according to tumor stage (B). The same data were stratified according to ER status (C). Each point represents the average of two technical repeats. Dots are individual samples; the lines refer to the median value. P-values (*P < 0.05, **P < 0.01, ***P < 0.001, and ****P < 0.0001) were measured by Mann–Whitney U test.

We tested the expression levels of E2F6 by qPCR on an array of 43 breast adenocarcinoma cDNAs and 5 normal breast cDNAs. Using primers common to all E2F6 transcript variants, high levels of cDNA were found in the tumor samples relative to the normal tissue. Similarly, the same samples showed significant levels of E2F6 variant a. However, variant b was significantly underexpressed (Figure 3A; Mann–Whitney U test). Comparing the E2F6 cDNA levels at different tumor stages (Figure 3B) revealed each stage to have significantly more cDNA than normal tissue, especially when using E2F6 common primers (P < 0.001; Mann–Whitney U test), with a trend to suggest that expression may increase further as the tumor progresses. We also examined expression in ER-positive compared to ER-negative samples, and importantly there was a significant difference between these groups (Figure 3C).

### 3.2. E2F6 is highly expressed in breast cancer cell lines

**Figure 4:**
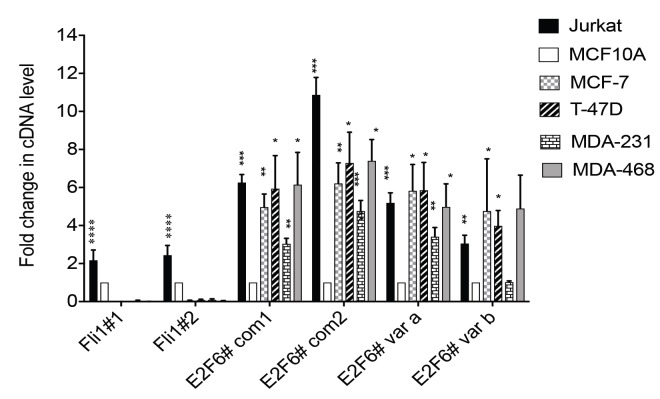
E2F6 cDNA levels were increased in breast cancer cell lines and Jurkat cells relative to MCF-10A. cDNAs prepared from cancer cell lines (MCF-7, T-47D, MDA-MB-231, MDA-MB-468, and Jurkat cells) and normal breast cells (MCF-10A) were subjected to qRT-PCR for E2F6. In this experiment we used four different primer pairs. The primer pairs (E2F6# com2, E2F6# var a, and E2F6# var b) are the same primers used in Figure 1. Primer pairs E2F6# com1 and E2F6# com2 detect a conserved region across E2F6 transcript variants, while primer pair E2F6# var a is designed to detect only E2F6 transcript variant a and primer E2F6# var b detects only E2F6 transcript variant b. In each case expression was first normalized to 18S rRNA. The level of each cDNA is shown relative to that of the same gene in MCF-10A (fold change). FLI1 cDNA level was checked using primers Fli1#1 and Fli1#2 as a negative control. Mean expression and error bars representing the corresponding standard deviation of 5 independent repeats are shown. The stars represent the P-values for the significant difference between the normal and cancer cell lines using unpaired Student t-tests.

**Figure 5:**
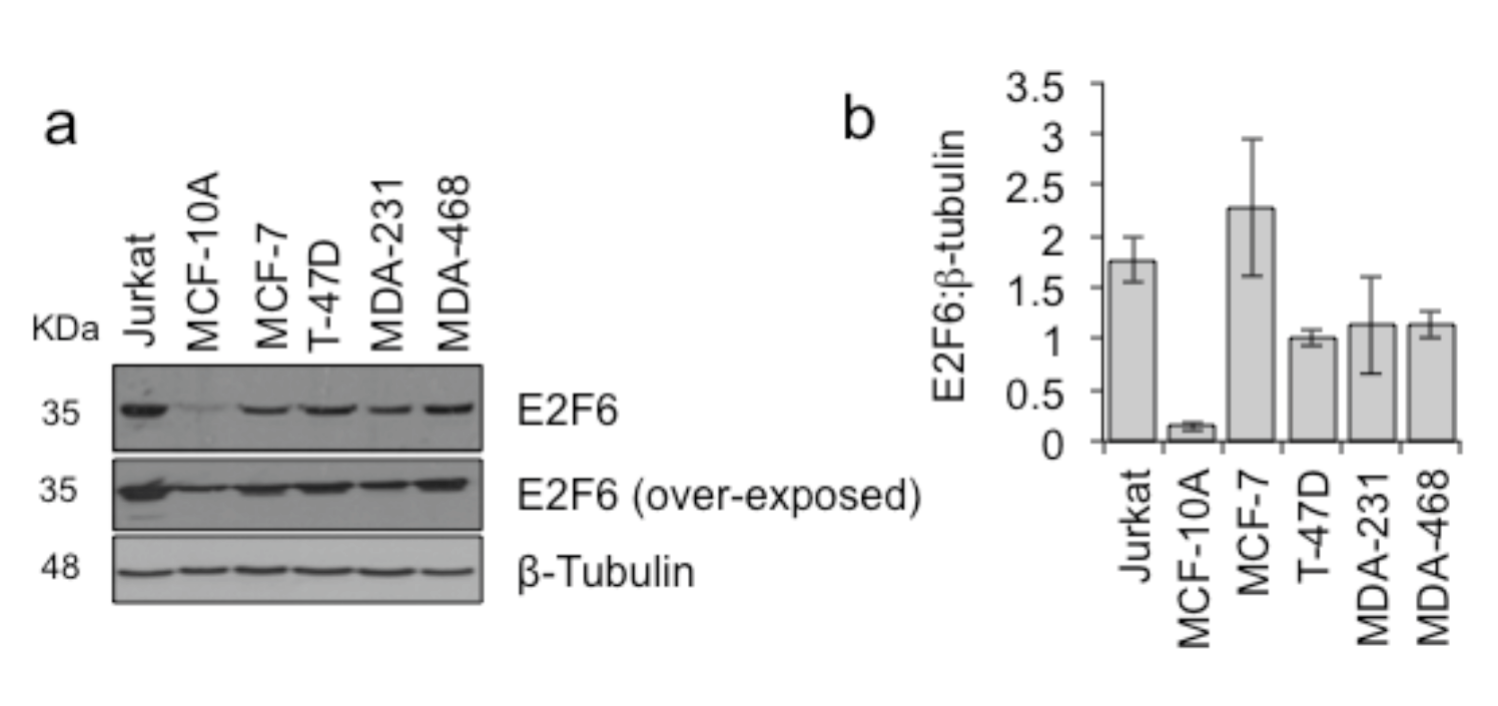
E2F6 protein levels are increased in breast cancer cell lines compared to normal tissue. Protein lysates from breast cancer cell lines (MCF-7, T-47D, MDA-MB-231, and MDA-468), Jurkat cells and nontumorigenic breast cells (MCF-10A) were western-blotted for detection of E2F6 and β-tubulin. (a) Representative example of western blots, (b) E2F6 protein levels quantified relative to β-tubulin. Mean and standard deviation of 4 independent repeats are shown.

In order to establish a system to investigate the importance of E2F6 overexpression, we determined whether or not the high expression of E2F6 mRNA from the tumor array was also seen in breast cancer cell lines. qRT-PCR analysis demonstrated that E2F6 mRNA is more highly expressed in breast cancer cell lines than in a nontumor breast epithelial cell line (Figure 4). The amount of E2F6 protein present was then assayed in a range of cell lines using MCF-10A to represent noncancer breast cells and using Jurkat cells known to overexpress E2F6 as a positive control. In the four breast cancer cell lines studied, we found that levels of E2F6 protein were higher compared to MCF-10A cells (Figures 5a and 5b). 

### 3.3. Overexpression of E2F6 is important to breast cancer cell viability

**Figure 6:**
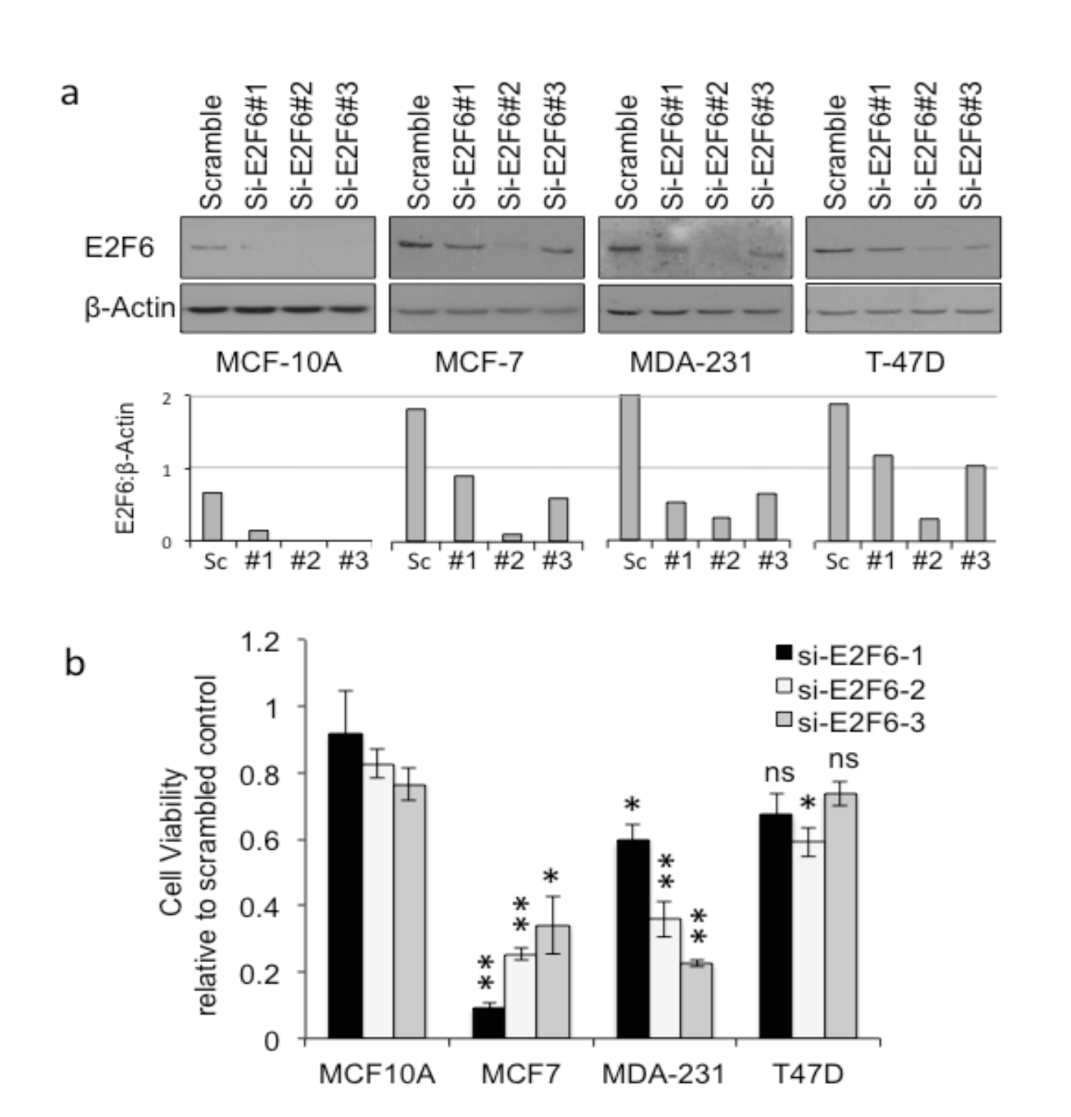
Depletion of E2F6 selectively kills breast cancer cell lines compared to normal cells. Normal (MCF-10A) and breast cancer (MCF-7, T-47D, MDA-MB-231) cell lines were transfected with scrambled si-RNA or one of three si-RNAs against E2F6 (si-E2F6#1-3). (a) Western blot confirmed E2F6 depletion after 48 h. Graphs represent protein quantification of E2F6 normalized to β-actin. (b) Cell viability relative to scrambled si-RNA transfected control as measured by MTT assay 96 h after si-RNA transfection. Means and standard errors of means of at least three independent experiments are depicted. Statistical significance (*P < 0.05, **P < 0.01, or ns - not significant) was calculated using a 2-tailed paired Student t-test comparing viability in each breast cancer cell line following each E2F6 si-RNA to the same si-RNA treatment in MCF-10A.

To determine whether or not the high expression levels of E2F6 in breast cancer cell lines were important for their survival, we set out to reduce protein levels and looked for any impact on viability. Three si-RNAs were designed to be specific to E2F6 (si-E2F6) and one scrambled si-RNA served as a negative control. In MCF-10A cells, for which expression was already low compared to the tumor cell lines, E2F6 became almost undetectable after transfection with each of the specific si-E2F6s (Figure 6a). In the tumor cell lines, the impact of transfection varied among the three si-E2F6s, but in all cases there was a reduction in detectable E2F6 compared to scrambled si-RNA.

The MTT assay was used to determine cell viability following transfection with the three si-E2F6s compared to transfection with scrambled si-RNA (Figure 6b). The viability of all three breast cancer cell lines was reduced by E2F6 depletion, while the MCF-10A control cells remained viable. Among the tumor cells lines, MCF-7 cells were the most sensitive to E2F6 depletion and T-47D was the least affected, with no significant difference in viability for two of the si-E2F6 knockdowns in that cell line. In T-47D cells, where survival was not significantly affected, protein depletion was also less efficient. Thus, like MCF-7 and MDA-MB-231 cells, T-47D cells may also be susceptible to a reduction in E2F6 expression if E2F6 levels drop below a certain threshold. This was clear upon transfecting T-47D cells with si-E2F6#2, which caused a severe decrease in the level of E2F6 leading to significant cell death. 

### 3.4. Cell death from reduced E2F6 levels during G1 or S-phase of the cell cycle

**Figure 7:**
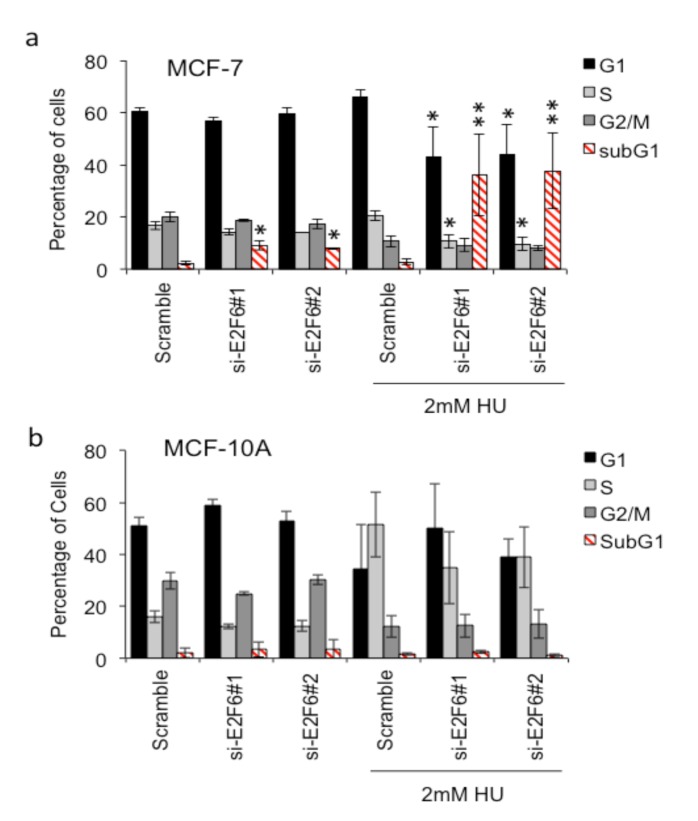
Replication stress increases dependency on E2F6 in breast cancer cells: (a) breast cancer (MCF-7) and (b) normal (MCF- 10A) cell lines were transfected with scrambled, si-E2F6#1, or si-E2F6#2 si-RNA. Twenty-four hours after transfection 2 mM HU was added to the cells, which were left for a further 24 h prior to cell cycle analysis by propidium iodide staining. Mean and standard deviation of three independent experiments is shown. Statistical significance was calculated using the paired Student t-test, comparing the proportion of cells in each stage of the cell cycle

The previous section tested in the importance of E2F6 expression on the viability of unperturbed exponentially growing cell cultures. Fluorescence-activated cell sorting (FACS) analysis was used to indicate at which stage of the cell cycle death might be occurring (Figure 7; Supplementary Figure 1). Consistent with the MTT results, we noticed that si-E2F6 caused a small but significant increase in the proportion of cells with sub-G1 DNA content in MCF-7 cells but not MCF-10A cells. However, no significant alteration in cell cycle profile was observed. 

Then we considered that E2F6 might have a role in responding to endogenous replication stress in breast cancer cells. To test this idea, we looked at the effect of E2F6 depletion on cell cycle profiles and cell death following exposure to HU to increase DNA replication fork stress. A level of HU was used that is considered to induce fork collapse and thus DNA damage in S-phase cells. We predicted that further replication stress in cancer cells would increase the proportion of cells with sub-G1 DNA content when E2F6 expression was reduced. Addition of HU 24 h after transfection with E2F6 si-RNA led to a large increase in the proportion of MCF-7 cells with sub-G1 DNA content (Figure 7a), consistent with an inability to stabilize and/or resolve perturbed replication forks. In contrast, the proportion of sub-G1 DNA content cells in MCF-10A cultures was unaffected despite the HU-induced increase in S-phase cells (Figure 7b). In addition, depletion of E2F6 combined with HU-induced replication stress in MCF-7 cells resulted in a reduced proportion of cells in G1 and S-phase but no increase in G2/M cells. This suggests that without E2F6, MCF-7 cells under increased replication stress are dying during G1 or S-phase. S-phase death seems more likely as this is where the HU-induced stress occurs.

## 4. Discussion

E2F6 is an important regulator of transcription, binding to the promoters of a wide range of genes (Oberley et al., 2003; Griagrande et al., 2004; Xu et al., 2007). Normally, it acts as a transcriptional repressor of E2F-responsive G1/S genes during the S-phase of the cell cycle (Griagrande et al., 2004; Bertoli et al., 2013). However, in cancer cells, E2F6 has been shown to play a dual role as a transcriptional activator and repressor (Xu et al., 2007). Here, we show that expression of E2F6 in breast tumor tissues and breast cancer cell lines is higher than in normal cells, supporting the view that E2F6 can be overexpressed in breast carcinomas (Palacios et al., 2005). Nevertheless, many studies have frequently exhibited conflicting E2F expression patterns and prognostic impacts, even in the same carcinoma type (Lu et al., 2004; Reimer et al., 2006; De Meyer et al., 2009). Recently, Li et al. (2018) investigated the mRNA expression patterns of E2Fs in breast cancer using Oncomine and The Cancer Genome Atlas (TCGA) data and found that E2F6 expression had no difference between tumor and normal tissues. 

In the current research, the expression of *E2F6 *splice variants was investigated. To our knowledge, this study is the first to look for the mRNA level of *E2F6* variants in breast cancer. Interestingly, the expression of the transcript variant *E2F6-a *was abundant in the breast tumor cDNAs, unlike variant *E2F6-b*,**which was underexpressed in almost all of the samples relative to the normal tissue. However, the breast cancer cell lines showed different expression pattern with regard to variant *E2F6-b*, whose level was higher than normal in the studied cancer cell lines except for MDA-MB-231. This discrepancy in *E2F6-b *expression between the tumor samples and the cancer cell lines can be interpreted according to the notion that says it is unclear how well tumor subtypes truthfully represent counterparts of cell line subtypes at the genomic level (Kao et al., 2009). While primary tumors frequently display numerical abnormalities, metastatic breast cancer cell types commonly show structural changes and amplifications (Willman and Ra, 2006). **

High expression levels of E2F6 have previously been associated with ER-negative/P53-positive breast carcinomas (Palacios et al., 2005) and triple-negative breast cancer, in which the negative regulator miR-185 is downregulated (Tang et al., 2014). Therefore, this study set out to analyze the expression of E2F6 in breast tumor and breast cancer cell lines and further test its correlation with ER level. Our data show overexpression of E2F6 in both ER-positive and ER-negative breast tumors compared to normal breast tissue, although the expression in the ER-positive samples was significantly higher than that in ER-negative. Concerning cancer cell lines, E2F6 was also found to be highly expressed in MCF-7 and T-47D cells (both are ER-positive) as well as MDA-MB-231 cells (ER-negative). 

Examining protein levels from breast cancer cell lines, we found high levels of E2F6 protein, as predicted from the cDNA levels found in tumors. Thus, these cell lines can be used as a good model for the study of E2F6 dependence in breast cancer. It has been indicated that overexpression of a particular protein in a cancer can indicate a dependence on that protein for tumor cell viability (Li et al., 2014). Whether this high expression of E2F6 is important to the viability of breast cancer cells was further tested in the present study using three specific si-RNAs targeting *E2F6* at various regions. Gene knockdown revealed varying degrees of depletion in the normal and cancer cell lines. Si-E2F6#2 was the best in decreasing E2F6 in all the studied cancer cells. However, all si-RNAs successfully depleted E2F6 in MCF-10A cells. Then the cell viability following si-RNA treatment was determined using the commonly used MTT assay, which is a colorimetric assay for assessing cell metabolic activity and can also be applied to measure cytotoxicity (loss of viable cells) as the dead cells lose their metabolic activity. While survival of MCF-7 cells was the most sensitive to knockdown of E2F6, T-47D cells, on the other hand, were the least influenced. Although these cell lines share mutual characteristics, i.e. both are luminal A (ER-positive, PgR-positive, and HER2-negative) (Kao et al., 2009; Holliday and Speirs, 2011), a difference between the cell lines is that the tumor type of MCF-7 cells is metastatic adenocarcinoma, while that of T-47D is invasive ductal carcinoma (Kao et al., 2009). From another point of view, looking carefully at the protein levels following treatment with si-RNAs suggests that more E2F6 protein remained present in T-47D cells. We suggest that the reduced impact on viability, compared to MCF-7 and MDA-231 cells, is because E2F6 remained above a critical threshold in T-47D cells. The viability of MDA-MB-231 cells was reduced upon E2F6 depletion. It is known that MDA-MB-231 is triple-negative, and its tumor type is metastatic adenocarcinoma (Kao et al., 2009). 

In contrast, MCF-10A cells, which are not cancer cells, remained viable even though the E2F6 protein levels were reduced the most. Thus, different sensitivities to si-E2F6 might be because E2F6 shows cell-type specificity and target-gene selectivity (Oberley et al., 2003). In agreement with this, E2F6 possibly targets different gene promoters in each of the studied normal and cancer cell lines; this needs to be extensively investigated using chromatin immunoprecipitation for further confirmation of this idea, at least in breast cancer. Oberley et al. (2003) compared the occurrence of E2F6 in the promoter of its target gene**(*ART-27*) between HEK293 and HeLa cells and found that the *ART-27 *gene promoter was enriched with E2F6 in the first cell line, but not in the second one, in spite of the higher levels of E2F6 protein being expressed in HeLa than HEK293 cells (Ogawa et al., 2002). Similarly, Xu et al. (2007) demonstrated that E2F6 targeted different genes in various cell types. In the same study, MCF-10A cells were found to have a very low number of E2F6 target genes. 

Overall, the above data show that depletion of E2F6 is deleterious in breast cancer cells with little effect in noncancerous breast cells, suggesting that E2F6 can become essential in highly proliferative tissue. These results are consistent with the evidence that overexpression of E2F family members correlates with metastasis and poor prognosis in breast cancer (Thangavelu et al., 2017). Likewise, many members of the E2F family with increased expression have been found to be significantly correlated with decreased overall survival in breast cancer patients (Li et al., 2018).

Recently, a mathematical model on how E2F6 functions to promote ovarian cancer stemness has been described. It has been shown that treatment of immortalized ovarian surface epithelial cells with estrogen upregulated E2F6,**which, in turn, competitively inhibited the activity of microRNA-193a (which usually prevents cancer stemness), thereby stimulating ovarian cancer stemness and tumorigenesis (Cheng et al., 2019). However, another route through which E2F6 may work was suggested to be competing with E2F1-3, which promotes cell cycle progression and apoptosis (Muller et al., 2001). Cancer cells are known to have higher levels of replicative stress than nontumorous cells (Macheret and Halazonetis, 2015). Recent work has demonstrated that E2F-dependent transcription also allows cells to tolerate such replication stress (Bertoli et al., 2016), reducing levels of DNA damage and stabilizing replication forks. In the present study, we considered it possible that E2F6 plays a role in responding to endogenous replication stress in breast cancer cells. Consequently, in response to replication stress, CHK1 phosphorylates E2F6, preventing binding to E2F promoters and thus allowing transcription of hundreds of genes important for protecting the genome, including replication fork integrity factors (Xu et al., 2007; Bertoli et al., 2013). It is thus suggested to function as an essential negative feedback mechanism during replication stress. 

From FACS analysis we determined that MCF-7 cells treated with si-E2F6 accumulate a higher proportion of sub-G1 cells than the scrambled control. Treating the cells with HU caused a significant (more than 2-fold) further increase of the proportion of cells with sub-G1 DNA content. Moreover, E2F6 depletion combined with HU-induced replication stress in MCF-7 cells caused a decrease in cell proportions in G1 and S-phase but no increase in G2/M cells. This suggests that MCF-7 cells devoid of E2F6 and under increased replication stress are dying during G1 or S-phase. Death in the S-phase seems more likely as this is where the HU-induced stress occurs. The current study proposes that in MCF-7 cells depleted of E2F6, replication forks may collapse, resulting in DNA damage and inducing cell death in the S-phase. In cancer cells, E2F6 overexpression might be favored as a bypass mechanism during replicative stress, facilitating increased proliferation. This idea is supported by our finding that HU-treated MCF-7 cells (expressing E2F6) do not accumulate in the S-phase, whereas MCF-10A cells do. Furthermore, we do not fully understand the oncogenic specific contribution to replication stress, and it is possible that cell line-specific differences may contribute to the response. Therefore, our data further highlight the importance of the E2F pathways in response to replication stress and the need for increased understanding of this pathway. 

Taken together, the above data suggest that in breast cancer cell lines, E2F6 activity during endogenous and induced replication stress helps the cells avoid apoptosis, supporting the idea that in vivo E2F6 is oncogenic. In conclusion, these findings raise the possibility that E2F6 could be developed as a therapeutic target in cancers, where its expression is essential.

## Acknowledgments

This study was funded by the Higher Committee for Education Development in Iraq (HCED), Office of the Prime Minister, International Zone, Baghdad, Iraq. This work was also supported in part by Breast Cancer Now [grant number PR016], a CRUK grant [number C5759/A17098], and BBSRC BB/K009346/1.****Many thanks to Professor Alastair S.H. Goldman, University of Bradford, UK, as well as Senior Lecturer Helen Bryant, University of Sheffield, UK, for their guidance and support during this study.
